# Study on KAL1 Gene Mutations in Idiopathic Hypogonadotropic Hypogonadism Patients with X-Linked Recessive Inheritance

**Published:** 2015

**Authors:** Atefeh Ahmadzadeh, Elahe Ghods, Majid Mojarrad, Robab Aboutorabi, Mojgan Afkhamizadeh, Shokoofeh Bonakdaran, Zohreh Mosavi, Seyed Morteza Taghavi, Mohammad Hassanzadeh Nazarabadi

**Affiliations:** 1 *Department of Medical Genetics, Medical School, Mashhad University of Medical Sciences, Mashhad, Iran.*; 2 *Medical Genetics Research Center, Mashhad University of Medical Sciences, Mashhad, Iran.*; 3 *Department of Endocrinology Ghaem Hospital, Mashhad University of Medical Sciences, Mashhad, Iran.*

**Keywords:** Idiopathic hypogonadotropic hypogonadism, kallmann syndrome, *KAL1* gene, X-linked recessive, GnRH

## Abstract

Idiopathic hypogonadotropic hypogonadism (IHH) is a condition caused by low doses of hypothalamic gonadotropin-releasing hormone (GnRH) leading to absence or incomplete sexual maturation. One of the disorders leading to IHH is Kallmann syndrome which is characterized by GnRH deficiency with anosmia or hyposmia. This disorder generally occurs as a hereditary syndrome with X-linked recessive inheritance pattern. However, autosomal dominant or recessive and sporadic cases have also been reported. *KAL1* is the most common mutated gene among these patients. The aim of this study was to determine the mutation spectrum of *KAL1* gene in twenty patients. KAL1 exons were amplified by PCR method and the products were assessed by high resolution melting (HRM) technique. In addition, for one of the patients, all coding exons of the *KAL1* gene were sequenced. Deletion of exons 4, 5 and 6 were evident in 5%, 10%, and 10% of patients, respectively. Furthermore, HRM results showed hemizygous mutation of exon 12 with more than 95% probability in 25% of patients. Finding these mutations could be helpful in the early diagnosis and presymptomic treatment of Kallman syndrome.

Idiopathic hypogonadotropic hypogonadism (IHH) is characterized by delayed sexual development as well as infertility caused by gona-dotropin- releasing hormone (GnRH) produc-tion deficiency from the hypothalamus (1, 2). IHH affects about 1 per 10,000 male and 1 in 50,000 female (1).

Hypogonadotropic hypogonadism (HH) can occur as either acquired or congenital form. Acquired HH may be caused by a variety of environmental factors such as drugs (for example: sex steroids and gonadotropin- releasing hormone analogues), head trauma, infiltrative or infectious pituitary lesions, endocrinopathies such as hyper-prolactinemia, pituitary or brain radiation, exhaus-ting exercise, and alcohol abuse.

The congenital HH is divided into two subtypes based on the presence of the sense of smell, which consist of congenital normosmic isolated HH (IHH) and anosmic HH or Kallmann syndrome (KS) (3).

KS may have a wide variety of clinical manifestations such as underdeveloped or absent olfactory bulbs, optic atrophy, deafness, cleft lip, renal malformations, cryptorchidism and neurological anomalies (4). KS constitutes approximately two-thirds of congenital HHs (3). This syndrome shows a wide genetic heterogeneity since various modes of inheritance, including: X-linked recessive, autosomal recessive, autosomal dominant as well as sporadic condition have been observed (5). Several genes have so far been shown to be involved in disease occurrence including *KAL1*, *FGF8*, *FGFR1*, *PROK2*, *PROKR2 *and *WDR11* (4, 6). The criteria for the diagnosis of KS include absent or incomplete sexual puberty (hypogonadism), anosmia or hyposmia, infertility (almost all untreated patients are infertile), decreased muscle strength and diminished aggressiveness and drive (in men), lack of breast development in women, small penises (<8 cm long in adults) as well as low levels of serum testosterone (less than 100 ng/dL) and decreased prostate size in men.

*KAL1* is located at Xp23.3 and is the most common mutated gene causing KS in 10% of patients (4, 5). This gene encodes for anosmin-1 which is an embryonic component of the extracellular matrix and is involved in GnRH induced olfactory neurons migration from the olfactory placode to the hypothalamus during embryonic life (5, 7-9). Absence of olfactory nerve cells extension to the olfactory bulb will impair or suppress the sense of smell (4, 5, 10, 11). GnRH controls the production of several hormones leading to sexual development before birth and during puberty (4).

Mutations in *KAL1* also induce severe reproductive phenotypes including absent puberty and high frequency of cryptorchidism or microphallus. The aim of this study was to investigate *KAL1 *mutations among Iranian patients.

## Materials and methods

**Patients**

Twenty unrelated patients including 12 females and 8 males, aged from 15 to 29 years old suffering from KS were referred to endocrine research center (MUMS) and were enrolled into this study. Patients with the existence of secondary sexual and abnormal MRI were excluded from the study.

The study design was approved by the Ethics Committee of Mashhad University of Medical Sciences and informed consent was obtained from patients.

**Molecular analysis**

Five ml of peripheral blood was collected into EDTA containing tubes. DNA was extracted according to Enghelabifar et al. (12). Each of 14 exons of the *KAL1 *gene was amplified by separate PCR reaction using specific primers listed in table 1. PCR was performed in 25 µl reaction containing 1X PCR buffer, 1-2 mM MgCl2, 0.2-1 mM of each dNTPs, 0.4 μM of each primer, 1-2.5 units of Taq DNA polymerase and 100 ng of template DNA. PCR reactions were performed in 2720 thermal cycler (Applied Biosystems, USA) with a cycling program of initial denaturation at 95 ºC for 5 min followed by 30-35 cycles of denaturation at 95 ºC for 30 s, annealing at 63 ºC for 30 s and extension at 72 ºC for 50 s; and a final extension step at 72 ºC for 5 min. PCR products were electrophoresed on 2% agarose gel and were visualized under UV light after ethidium bromide staining.

PCR products were also screened for mutation using high resolution melting (HRM) experiments using evagreen HRM mastermix. Melting analysis was performed by Q- Rotor instrument (Qiagen, Germany) by heating samples gradually from 50 ^o^C toward 95 ^o^C, 0.1 ^o^C each step.

(F=Forward and R=Reverse) Fluorescent detection lasted 10 s in each step.

**Table 1:** PCR primer sequences

Exon	Primer Sequences 5' 3'	Product size (bp)
EX1	F TTGAACTTTCCGGCTCAGTC	363
	R GCAGCCCCAGAAAGAACC	
EX2	F GTGTAGCTTTCTAATGGATCA	218
	R ATTGGTGGAAACTGGGCATA	
EX3	F CAGGCATTGAAAAAGCAACA	225
	R TGACCCCACGTAAGCATAGTC	
EX4	F TCAGACTTCATGTGTCTTTAATGGA	345
	R CTTCCCTAGGCACACACAGA	
EX5	F GTTCTTCCTCAACTTTTACTTCA	303
	R CAGACACTACCTCCAGGATGA	
EX6	F GATCCAACTAACATGTCGGAAT	250
	R GTGTGCCTGGTAGCAAGGAT	
EX7	F CAATGCTTCACGTGTTGACC	340
	R CCCTCTGTGGGAATAACAATC	
EX8	F TTGCAATGAAGATGAGAGACG	285
	R CTCCATTGTGCCTTGTTGTG	
EX9	F CCATCTTGCCCAGGAATCTA	299
	R TGGCTTGACATTTACTTCTTCAAA	
EX10	F TACCTGGAATGTAACATCCA	278
	R CTTCCATCAAGTCATTACTCC	
EX11	F ATCCTTGTTGGATGGAATATG	349
	R TGAAACGCAGTTTGACAAGG	
EX12	F ACACCTTCTCCAGTCGCCTA	319
	R AGCAGTAGATACCAATGACACA	
EX13	F CGGTGAGCATGCTCTTTTATG	232
	R TTTCTCTATGTCCACAAGACCTG	
EX14	F AGGAACATTTGCCAGGGTCT	245
	R TCTGGAAGTGTGCATGTCTC	

**Fig. 1:**
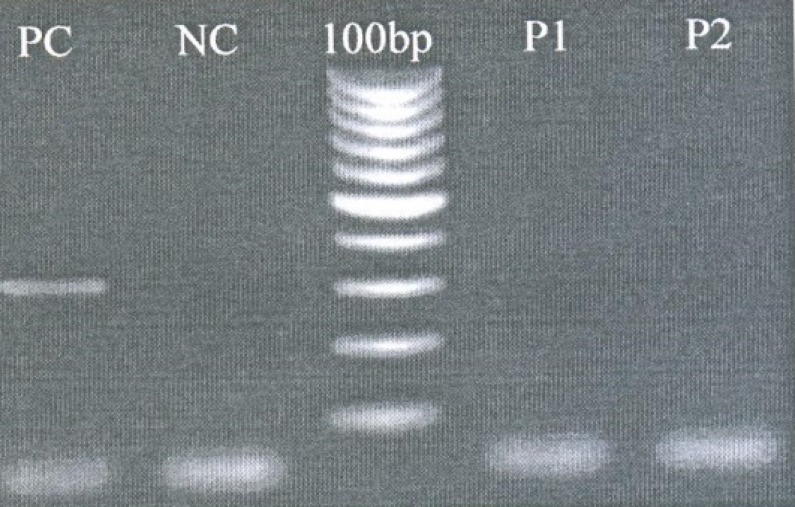
Representative agarose gel (2%) indicating Exon 5 deletion. Length of presented band is 303 bp. 100bp DNA size marker was used to estimate PCR product bands. PC= Positive control. NC= Negative control. P1= Patient no 1. P2= Patient no 2

**Fig. 2:**
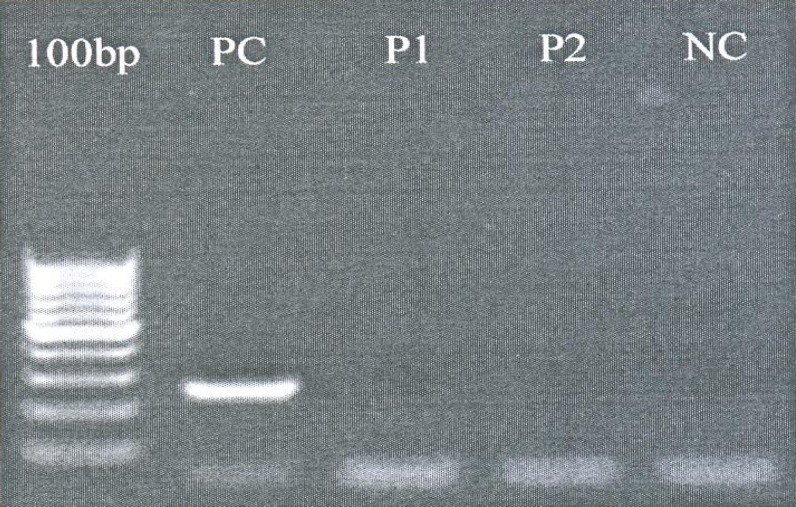
Representative agarose gel (2%) displaying Exon 6 deletion. Length of presented band is 250 bp. 100 bp DNA size marker was used to estimate PCR product bands. PC= Positive control. NC= Negative control. P1= Patient no 1. P2= Patient no2

**Fig. 3:**
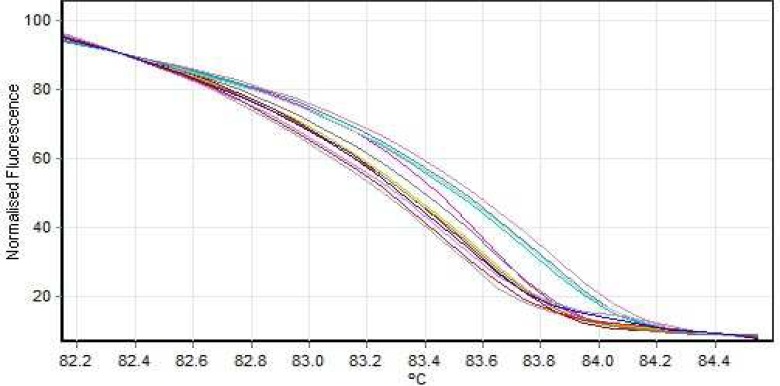
Normal analysis HRM graph exon no 2

**Fig. 4:**
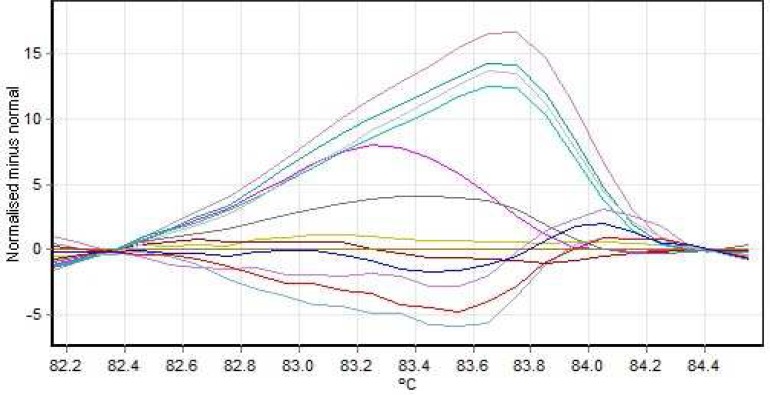
Normal graph exon no 2 (median line= no mutation, ascending line= homozygous, descending arc= heterozygous

**Fig. 5:**
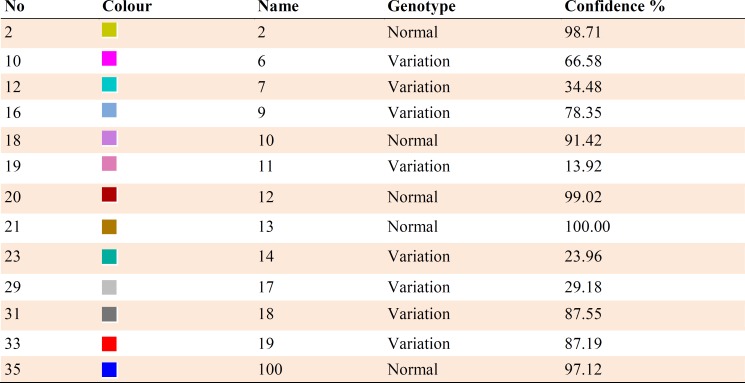
Hemizygous mutation of exon 12

**Fig. 6:**
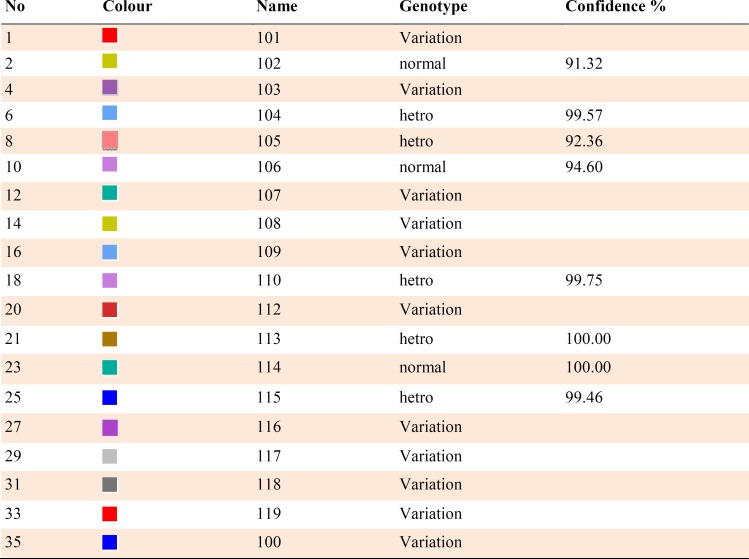
heterozygote mutation of exon 12

**Fig.7:**
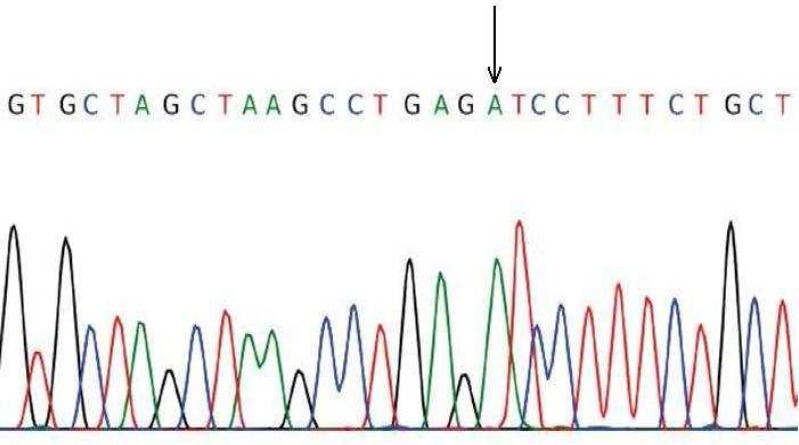
Normal graph exon no 12 (median line= no mutation, ascending line= homozygous, descending arc= heterozygous

Data were normalized against heterozygous control samples. Samples with more than 90% probability of presence of mutation were considered as positive results.

In addition, all exons of the gene were sequenced in one patient, using Sanger dideoxy method and were analyzed through Chromas software.

## Results

**Molecular detection of exon deletions by PCR**

Upon PCR andysis we detected 5 male patients with hemizygous mutation. Deletion in exon 4 (1 case), exon 5 (2 cases) (figure 1), as well as complete deletion of exon 6 (2 cases) were detected as three distinct abnormalities (figure 2).

**High resolution melting (HRM) analysis**

HRM reaction was performed for all patients using specific primers of all 14 exons. In all reactions, negative controls were applied in order to exclude any contamination. However, no point mutation was evident (figures 3, 4; table 2).

After sequencing, Chromas software allowed the detection of a hemizygous point mutation in exon 12 of *KAL1* gene causing a change from Asn (AAC) to Ile (ATC) in codon 623 (figure 5). Four patients from both genders had heterozygous or hemizygous mutation in exon 12, at this position with a probability higher than 95% according to HRM analysis (figures 6, 7; table 3)

**Table 2:** Color, name = patient code, genotype, and confidence % of analysis of samples for exon 2.

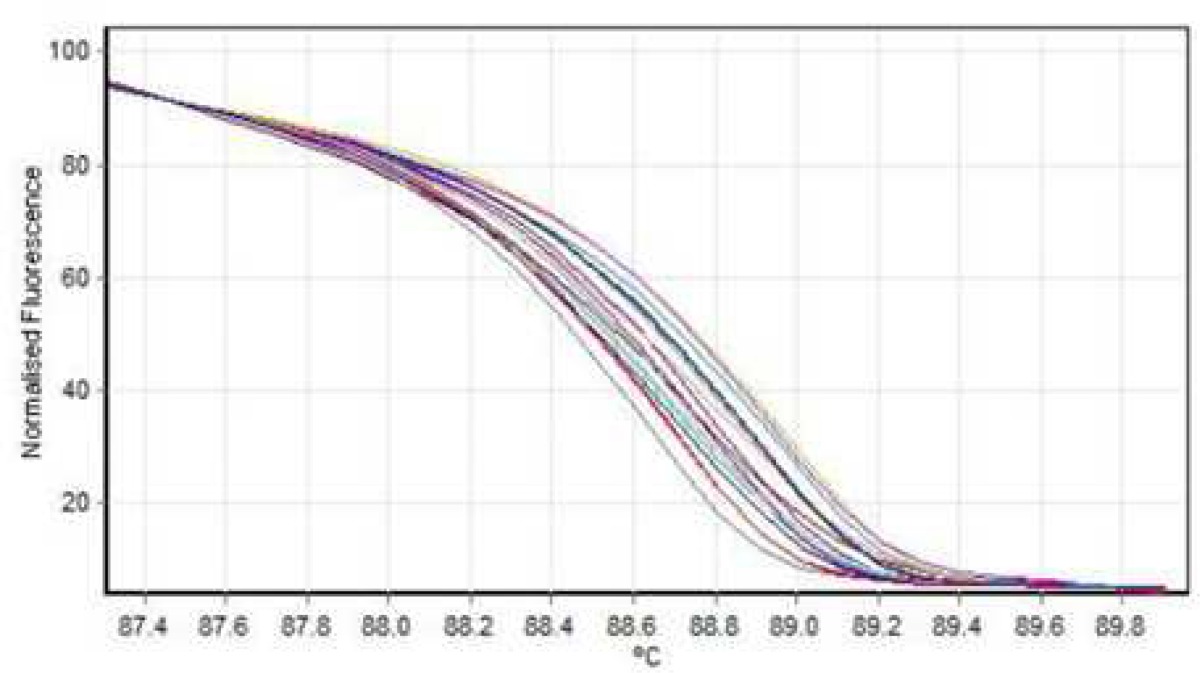

**Table 3:** Color, name = patient code, genotype, and confidence % of analysis of samples for exon 12

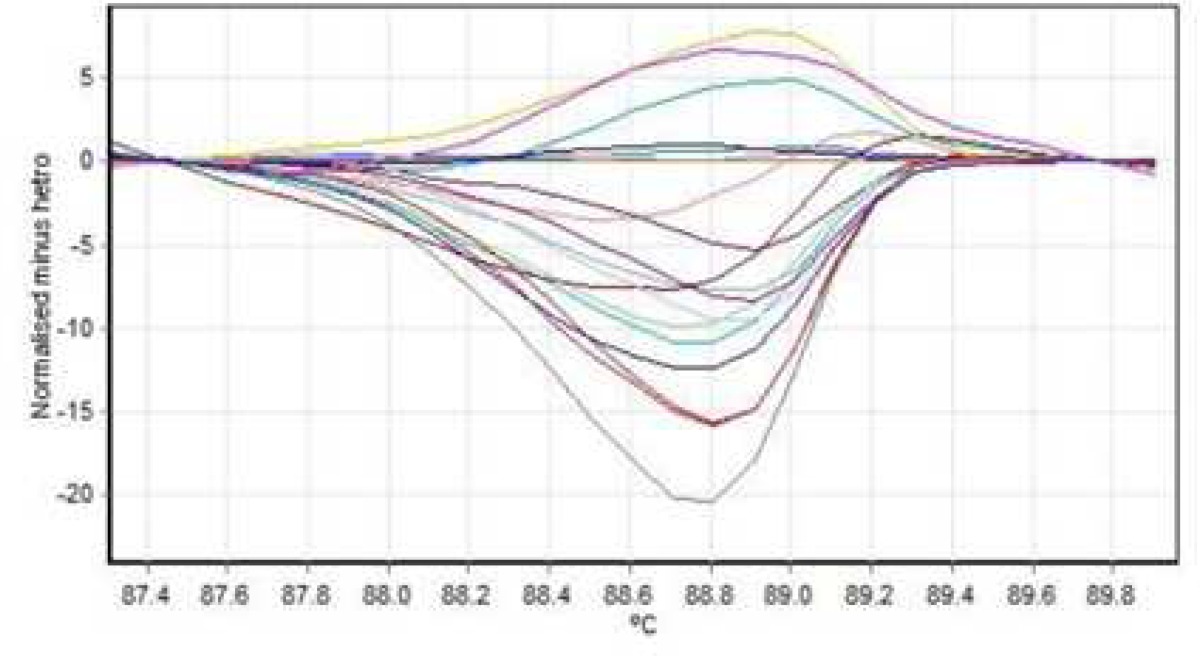

**Table 4:** Mutations identified in Sato et al. study

*KAL1* mutation	Nucleotide change	Amino acid change	position
One missense	488G>A	C163Y	Exon 4
Three nonsense	1270C>T	R424X	Exon 9
	1891C>T	R631X	Exon 13
	571C>T	R191X	Exon 5
Three frameshift	100–101del CG	---	Exon 1
	262–269delGAGCCCTG	---	Exon 3
	714–715del GA	---	Exon 5
Splice donor site	IVS4+1G>T	Splicing error	Exon 4

## Discussion

KS may be caused by mutations in * KAL1*, *FGFR1*, *PROKR2* and *PROK2* genes leading to types 1 to 4 KS respectively, among which type 1 is the most common form. Due to adverse conditions such as absent puberty, infertility and the impact of these conditions on the affected person’s social and health life, investigation on this disease is important for the diagnosis and treatment of patients before clinical manifestations.

In the present study, twenty patients with X-linked pattern of inheritance were investigated for *KAL1* gene mutations. The molecular study showed that exonic deletions constituted almost 25% of genetic etiology of X-linked KS patients analyzed.

Deletion of exon 4 occurred within the region encoding the whey acidic protein domain of the KAL1 protein, which is involved in axonal targeting process (13). Deletions in exons 5 and 6 occurred within the region encoding the first fibronectin type III- like repeat of the KAL1 protein, which is involved in neuronal migration and axonal targeting processes (14). These mutations are different from those reported previously in other populations. In 2004 Sato et al. studied twenty -eight KS patients by whole exome sequencing of *KAL1* gene and detected eigth point mutations. The mutations consisted of one missense, three nonsense and three frameshift mutations and one splice donor site (table 4) (15).

Laitinen et al. in 2011 investigated thirty patients affected with KS. They analyzed 7 identified KS genes (*KAL1*, *FGFR1*, *FGF1*, *PROK2*, *PROKR2*, *CHD7* and *WDR11*). But only three male patients showed mutation in *KAL1* gene, whereas, nine patients had mutation in *FGFR1* gene. *KAL1* mutations were nonsense mutation R262X, the frameshift mutation S158WfsX45, and a deletion of the last nucleotide of exon 8 and the first three nucleotides of intron 8 (g.2357_ 2360delAgta) abolishing the splice site (16). The results of this study was in contrast with the present investigation. This difference can be due to the selection of patients, with X-linked recessive pedigree in our cases, while Laitinen et al. investigated KS patients with different inheritance patterns.

Newbern et al. in 2014 identified a mutation in homeobox gene expressed in embryonic stem cells *(HESX1)* involved in HH including incomplete forms and also the incomplete forms of KS (17).

According to the present study, molecular analysis of *KAL1* gene can be helpful for the diagnosis of X- linked recessive KS. Detection of mutation in these conditions can help presympto-matic diagnosis of members and therfore facilitate early therapies. Further exome sequencing of other patients should help to find other molecular defects in this gene.
